# MRI findings of an incidental ectopic posterior pituitary gland in an adult: Case report and review of T1 hyperintense lesions in the literature

**DOI:** 10.1016/j.radcr.2026.03.050

**Published:** 2026-05-02

**Authors:** Salma Hashem, Roméo Bujiriri Murhega, Patient Aganze Migabo

**Affiliations:** a Department of Radiology, Gonesse Hospital, France; b Faculty of Medicine, Hôpital Provincial Général de Référence de Bukavu (HPGRB), Université Catholique de Bukavu (UCB), South Kivu, Democratic Republic of the Congo

**Keywords:** Ectopic posterior pituitary, Magnetic resonance imaging, Incidental finding, Suprasellar, Pituitary stalk interruption syndrome

## Abstract

The ectopic posterior pituitary represents a rare congenital malformation attributable to aberrant embryogenesis of the neurohypophysis. Although it may occur as an isolated anomaly, it is frequently concomitant with other congenital malformations of the central nervous system. Despite extensive investigations, the precise etiology remains elusive; however, accumulating evidence suggests that its pathogenesis may parallel that of septo‐optic dysplasia, thereby implicating a genetic predisposition. Earlier accounts predominantly attributed this anomaly to traumatic mechanisms. In contrast, contemporary studies increasingly support a hereditary basis. In this report, we delineate the magnetic resonance imaging (MRI) characteristics of an isolated ectopic posterior pituitary, incidentally discovered during an ataxia work-up in a 72-year-old female patient. Notably, the patient exhibited no clinical evidence of growth hormone deficiency or hyperprolactinemia.

**Key message**

Ectopic posterior pituitary is a rare congenital anomaly resulting from aberrant neurohypophyseal embryogenesis. Although frequently associated with other central nervous system malformations, it may be incidentally detected in the absence of endocrine dysfunction. Recognition of its distinctive MRI features is essential for accurate diagnosis and appropriate management in clinical practice.

Alt-text: Unlabelled box dummy alt text

## Introduction

Ectopic posterior pituitary gland (EPPG) is a rare congenital abnormality caused by a disruption in the normal development of the posterior pituitary during embryogenesis. It is most commonly identified in childhood and is frequently associated with pituitary hormone deficiencies, particularly growth hormone deficiency, as well as other congenital anomalies of the central nervous system [1,2].

Isolated EPPG without associated endocrine dysfunction is uncommon, particularly in adults. The diagnosis is made based on the findings obtained from magnetic resonance imaging (MRI), which is the only imaging modality capable of adequately identifying an ectopic posterior pituitary [3,4].

In the present article, we report the MRI findings of an isolated ectopic posterior pituitary gland, discovered incidentally during a work-up for ataxia in a 72-year-old woman with no signs of growth hormone deficiency (pituitary dwarfism) or hyperprolactinemia. This case illustrates an unusual presentation of EPPG and accentuates the importance of recognizing its imaging features even in asymptomatic elderly patients.

## Case presentation

A 72-year-old woman with diabetes mellitus presented to the emergency department of Gonesse Hospital in France with a complaint of ataxia that had persisted for a duration of one month. All biological tests yielded inconclusive results, and the patient has no history of hypopituitarism. The patient has a history of normal childbirth, and physical examination revealed no height anomalies, as the patient’s height is 154 centimeters.

**Fig. 1:**
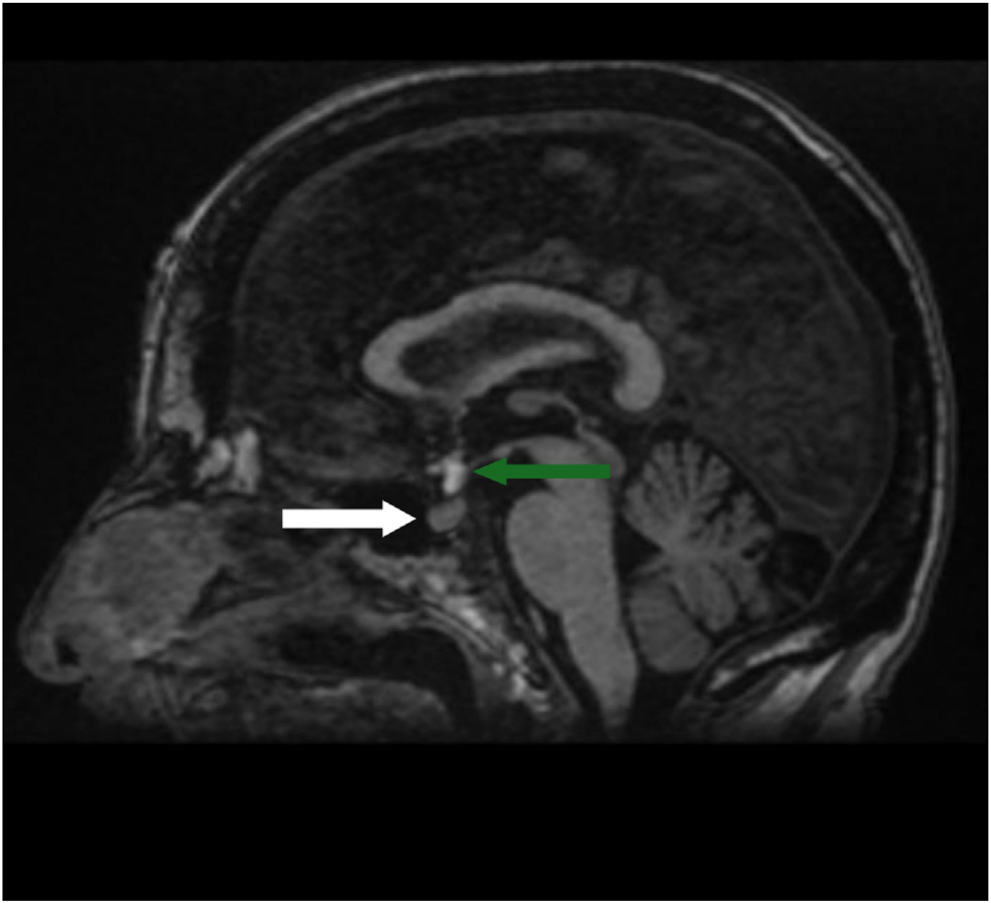
T1 midsagittal image slice showing a normal-sized homogenous pituitary gland (white arrow) in the sella turcica and a hyperintense signal suprasellar structure representing the ectopic posterior pituitary (green arrow).

**Fig. 2:**
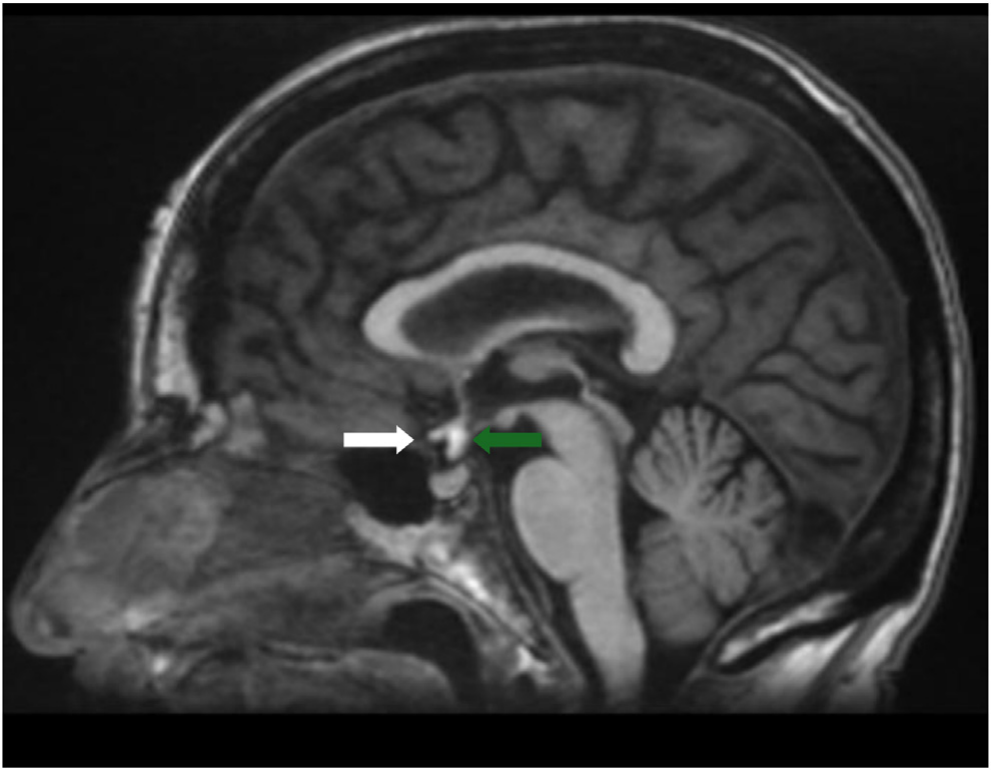
T1 left paramedian sagittal image showing the optic chiasm (white arrow) located anteriorly to the ectopic posterior pituitary gland (green arrow) without any mass effect.

**Fig. 3:**
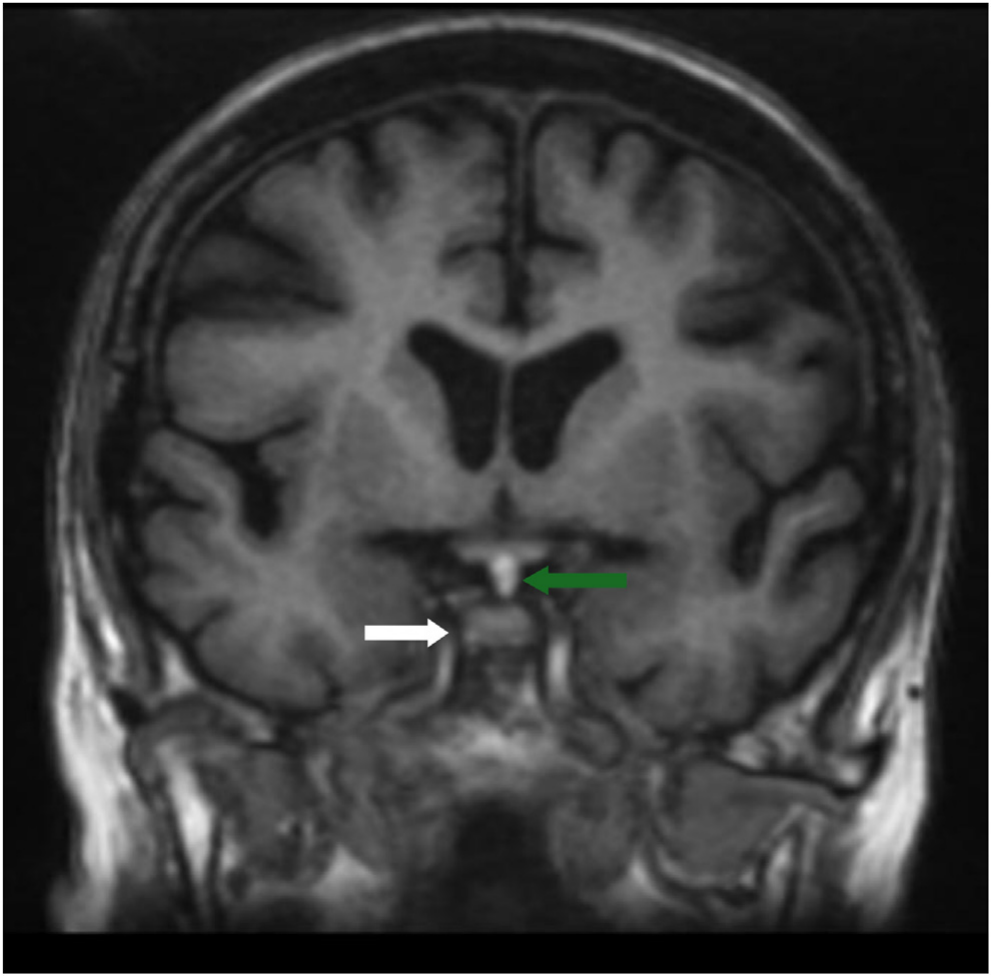
T1 coronal imaging slice through the pituitary gland (white arrow) showing the ectopic posterior pituitary gland (green arrow) at the median eminence.

For investigation, physicians requested a brain MRI to exclude cerebellar infarcts or other conditions related to this clinical presentation. A stroke protocol in addition to a 3D T1 sequence for brain morphology was performed with a General Electric 1.5 Tesla MRI machine: diffusion weighted image (DWI) and apparent diffusion coefficient (ADC), 3D T2 fluid-attenuated inversion recovery (FLAIR), T2 GE (gradient echo), and time of flight (TOF) were realized (Fig. 1, Fig. 2, Fig. 3).

The examination was negative for any acute infarct lesions, and we found only two chronic lacunar infarcts: in the left posterior temporal lobe and in the right cerebellar hemisphere, the latter was thought to account for the ataxia. No mass or bleeding was noted. We also observed moderate white matter T2 hyperintensities corresponding to chronic small vessel ischemia.

A T1 hyperintensity structure in the suprasellar region caught the attention, located in the region of the hypothalamus at the median eminence. We did not detect fatty or protein signals because dermoid cysts frequently occur in this region. The anterior commissure was located anteriorly with a normal size, appearing as an intermediate T1 hyperintensity. Furthermore, the sella turcica was within normal size, but no T1 hyperintensity was seen in the posterior zone. The anterior pituitary lobe was well seen, normal in shape and signal, but the pituitary stalk (infundibulum) was not identified. TOF images revealed non-aneurysmal lesions in the region. No associated malformations were identified. The patient underwent a head computed tomography scan 12 years ago, which was normal. Our analysis did not reveal any bone erosion in the sellar region nor an enlarged sella turcica.

## Discussion

EPPG has been mostly found in pituitary stalk interruption syndrome as part of the triad which associates: hypoplastic or aplastic anterior pituitary gland, a hypoplastic infundibulum and EPPG with a clinical manifestation of hypopituitarism. Therefore as a congenital pattern, the diagnosis is made during childhood and mostly during puberty [4,5]. Isolated findings have been reported, either associated with other malformations or not, and some with clinical manifestations [3,4,[6], [7], [8], [9]]. Since the analysis of the pituitary gland is one of the essential parts of brain MRI reports, radiologists should be aware of sellar and suprasellar anatomy to better assess malformations and normal variants, with the pituitary gland being the key of all hormones [10].

In the literature, several studies have discussed hypophyseal malformation and anatomical variants, mostly in childhood. To the best of our knowledge, cases of an incidental ectopic posterior pituitary gland in adults has been scarcely reported [4,[6], [7], [8]].

Trauma and post-surgery transsphenoidal process have been evoked among causes of EPPG along with some genetic mutations (LHX4, LHX4, OTX2, HEX1, SOX3, PROKR2, GPR161), with most cases being pediatrics [[11], [12], [13]].

The differential diagnosis of EPPG includes teratoma, dermoid cyst, and lipoma. MRI is the gold standard tool to assess sellar pathology, and T1 sequences in the sagittal plane are the best sequences. The pituitary-centered MRI protocol is the best, as it emphasizes the spatial resolution, allowing radiologists to better assess the morphology and skull base state. Sequences with fat suppression are not necessary, as we can exclude fat components by comparing them to the T2 signal which must be an isointense signal in EPPG tissue, but hyper or intermediate in malformations with fat composition [7,8,14].

Dermoid cysts remain a pattern that can challenge most radiologists due to their lipid (cholesterol) content, which shows a T1 hyperintense signal similar to that of the posterior pituitary gland. Therefore, the absence of a hyperintense signal in the posterior portion of the sellar region is the key to diagnosis. Furthermore, in T2 sequences, dermoid cysts tend to show heterogeneous signals. Being a space-occupying lesion, a dermoid cyst can exert a mass effect, and patients may present with diplopia or other neurological deficits by compressing the nerves in and around the cavernous sinus and Meckel’s caves (III, IV, V, VI cranial nerves) [14].

Radiologists and neurosurgeons should be aware of these findings to avoid unnecessary additional imaging, which can be costly and stressful for patients, since it may be an incidental finding without clinical manifestations.

Our patient has never had a craniocerebral trauma nor transsphenoidal surgery, and has never experienced hormonal disturbance, and she is currently old. Therefore, EPPG in this case could be considered as an anatomical variant, since we do not have any of the previously mentioned acquired potential causes, in addition to the absence of congenitally associated malformations such as pituitary stalk interruption syndrome, Kallmann syndrome, septo-optic dysplasia or corpus callosum agenesis [12,14,15].

## Conclusion

In conclusion, the incidental detection of an ectopic posterior pituitary on MRI underscores the importance of recognizing this rare congenital anomaly, even in the absence of overt endocrine dysfunction. The distinct imaging features observed in this case facilitate its differentiation from other sellar and suprasellar pathologies, thereby preventing misdiagnosis and unnecessary interventions. Notably, the patient did not require any treatment, highlighting that such incidental findings may not necessitate therapeutic intervention in the absence of clinical or hormonal abnormalities. This report emphasizes the need for further investigation into the genetic and embryological mechanisms underlying ectopic posterior pituitary development, as well as its potential long-term clinical implications such as abnormalities in stress hormone response, fluid imbalance in severe illness, or no manifestation in many cases. Enhanced awareness among clinicians as for endocrinologists and radiologists is essential to ensure accurate diagnosis and optimal patient management.

## Author contributions

P.M., the corresponding author, drafted the manuscript, including the case description and its conceptualization. R.M., co-author, was responsible for study conceptualization, writing and final validation. S.H., co-author, supervised the manuscript drafting, contributed to the editing and performed the final revisions. All authors approved the final version of the manuscript for publication.

## Ethical approval

This case report was conducted in accordance with the principles of the Declaration of Helsinki. As per institutional guidelines for case reports, formal ethical approval was not required.

## Data availability

The data supporting the findings of this case report are available from the corresponding author upon reasonable request.

## Patient consent

Written informed consent was obtained from the patient for publication of this case report and any accompanying images.
